# Serial changes of the side-branch ostial area after single crossover stenting with kissing-balloon inflation

**DOI:** 10.1007/s10554-023-02853-7

**Published:** 2023-05-16

**Authors:** Tatsuhiro Fujimura, Takayuki Okamura, Ryoji Nagoshi, Yoshinobu Murasato, Masahiro Yamawaki, Yosuke Miyazaki, Hideaki Akase, Shiro Ono, Takeshi Serikawa, Yutaka Hikichi, Hiroaki Norita, Fumiaki Nakao, Tomohiro Sakamoto, Toshiro Shinke, Junya Shite

**Affiliations:** 1 Division of Cardiology, Department of Medicine and Clinical Science, Yamaguchi University Graduate School of Medicine, 1-1-1 Minamikogushi, Ube, Yamaguchi, Ube, 755-8505 Japan; 2 Department of Cardiology, Osaka Saiseikai Nakatsu Hospital, Osaka, Japan; 3 Department of Cardiology, National Hospital Organization Kyusyu Medical Center, Fukuoka, Japan; 4 Department of Cardiology, Saiseikai Yokohama City Eastern Hospital, Yokohama, Japan; 5 Department of Cardiology, Saiseikai Yamaguchi General Hospital, Yamaguchi, Japan; 6 Department of Cardiology, Fukuoka Wajiro Hospital, Fukuoka, Japan; 7 Department of Cardiology, Saga-Ken Medical Center Koseikan, Saga, Japan; 8 Department of Cardiology, Oda Hospital, Kashima, Japan; 9 Department of Cardiology, Yamaguchi Grand Medical Center, Hofu, Japan; 10 Department of Cardiology, Saiseikai Kumamoto General Hospital, Kumamoto, Japan; 11 Division of Cardiology, Department of Medicine, Showa University School of Medicine, Tokyo, Japan

**Keywords:** Left main coronary artery bifurcation lesion, Percutaneous coronary intervention, Optical coherence tomography, Side-branch ostial area, Incomplete stent apposition

## Abstract

**Purpose:**

We aimed to investigate the serial change of the side-branch ostial area (SBOA) depended on the wire-position before Kissing-balloon inflation (KBI) in the single-stent strategy for bifurcation lesions separately in the left main coronary artery (LMCA) and in non-LMCA.

**Methods:**

Patients who underwent a single-stent KBI for a bifurcation lesion and had OCT images at the timing of the rewiring, at the post-procedure, and at the 9-month follow-up were extracted from the 3D-OCT Bifurcation Registry, which is a multicenter-prospective registry of patients with a percutaneous coronary intervention for a bifurcation lesion under OCT guidance. The SBOA was measured by dedicated software, and the rewiring position at the side-branch ostium after crossover stenting was assessed by three-dimensional-optical coherence tomography (3D-OCT). The optimal rewiring was defined as link-free-type and distal rewiring. The relationship between the optimal rewiring and the serial change of the SBOA was investigated separately in LMCA and non-LMCA cases.

**Results:**

We examined 75 bifurcation lesions (LMCA, n = 35; non-LMCA, n = 40). The serial changes of the SBOA with the optimal rewiring were not significantly different regardless of LMCA and non-LMCA (LMCA:3.96 to 3.73 mm^2^, p = 0.38; non-LMCA:2.16 to 2.21 mm^2^, p = 0.98), whereas the serial changes of the SBOA with the sub-optimal rewiring were significantly reduced (LMCA:6.75 to 5.54 mm^2^, p = 0.013; non-LMCA:2.28 mm^2^ to 2.09 mm^2^, p = 0.024). There was no significant difference in clinical events between the optimal and sub-optimal rewiring group regardless of the LMCA and non-LMCA.

**Conclusion:**

The side-branch ostial area dilated with the optimal rewiring position in a bifurcation lesion treated with single crossover stenting and kissing-balloon inflation was preserved regardless of whether the bifurcation was in the LMCA or a non-LMCA.

**Supplementary Information:**

The online version contains supplementary material available at 10.1007/s10554-023-02853-7.

Percutaneous coronary intervention (PCI) for bifurcation lesions remains one of the most challenging procedures in real-world practice because of the anatomical complexity and the various procedures and strategies involved. The use of intracoronary imaging is important in the PCIs of coronary bifurcation lesions. The superior resolution of optical coherence tomography (OCT) in particular provides potential advantages for the identification of the guidewire’s crossing point and stent optimization tools [1]. The performance of three-dimensional optical coherence tomography (3D-OCT)-guided PCI for bifurcation lesions can reduce the incidence of incomplete stent apposition at the bifurcation segment in patients who undergo single crossover stenting with kissing-balloon inflation (single-stent KBI) [2, 3]. Moreover, the side-branch ostial area (SBOA) was expanded even at 6–12 months post-intervention in single-stent KBI cases with link-free rewiring on the carina and distal rewiring to the side-branch identified by 3D-OCT [4]. However, that result was limited to retrospective data of a small patient series drawn from a single center.

PCIs for bifurcation lesions of the left main coronary artery (LMCA) are associated with a higher risk of target lesion failure compared to non-LMCA bifurcation lesions [5]. The impact of optimal rewiring under 3D-OCT guidance on the outcome of the side-branch ostial area in LMCA bifurcations, which have anatomic and prognostic characteristics that differ from those of bifurcations in non-LMCA, is unclear [6]. We conducted the present study to investigate the relationship between the optimal rewiring position at the side-branch ostium and the serial changes of the SBOA in single-stent KBI cases in a group of patients with LMCA bifurcation lesions and a group of patients with non-LMCA bifurcation lesions.

## Methods

We extracted the patients who underwent a single e-stent KBI for a bifurcation lesion and had assessable OCT images at the timing of the rewiring to the side branch before KBI, at the post-procedure (after completion of the procedure), and at the 9-month follow-up after the index PCI from a 3D-OCT bifurcation registry that enrolled patients who underwent a PCI for a *de novo* bifurcation lesion with ≥ 2-mm-dia. side-branches identified visually under OCT guidance. We excluded lesions with a poor OCT image in which a rewiring position in front of the side-branch ostium after main-vessel crossover stenting could not be assessed. The main vessel was defined as a stented vessel, and the bifurcation site was distinguished as the LMCA, the left anterior descending artery (LAD), the left circumflex artery (LCX), or the right coronary artery (RCA). For the present analysis, cases were divided into an LMCA group and a non-LMCA group including LAD, LCX, and RCA.

### QCA analysis

Quantitative coronary angiography (QCA) was performed using the QAngio® XA ver. 7.3 system (Medis Specials, Leiden, the Netherlands) pre-procedure, post-procedure, and at the 9-month follow-up. A bifurcation-dedicated tool was used for the analysis. The reference vessel diameter, percent diameter stenosis, late lumen loss, and bifurcation angle were measured at each site.

### OCT image acquisition and cross-sectional image analysis

OCT pullback images were acquired with a pullback speed of 18 mm/s or 36 mm/s using the ILUMIEN™ OCT Imaging system (St. Jude Medical, Minneapolis, MN, USA) and a Dragonfly™ intravascular imaging catheter. The stent area and lumen area in the main vessel were each measured at 1 mm intervals within 5 mm proximal and distal to the bifurcation area at the post-procedure and the 9-month follow-up, then the average of the stent area, lumen area, and neointima area were calculated.

Incomplete stent apposition (ISA), defined as a distance between the strut marker and the lumen counter that is greater than the specific strut thickness, was counted for every single strut on each frame at the bifurcation segment [7]. The ISA was assessed at the bifurcation area defined as an area from a carina point to a side-branch take-off point, and at the side-branch side and the opposite side that the bifurcation area was divided into two 180° halves toward or opposite of the side-branch at the post-procedure [8].

### 3D-OCT image analysis

The stent configuration and the rewiring position in front of the side-branch ostium were confirmed by 3D-OCT after main-vessel crossover stenting across the side branch and rewiring to the side branch before KBI as described previously [2]. Briefly, the link-connected type was defined as a case in which a link connected to the carina; the link-free type was defined as a case in which no link was observed at the carina. “Distal cell” was defined as a larger area enclosed by both the carina and the stent strut, with at least one distal top of the stent hoop located on the side-branch ostium. The optimal rewiring position was defined as the link-free type and distal rewiring, and the other cases were defined as having a sub-optimal rewiring position.

**Fig. 1:**
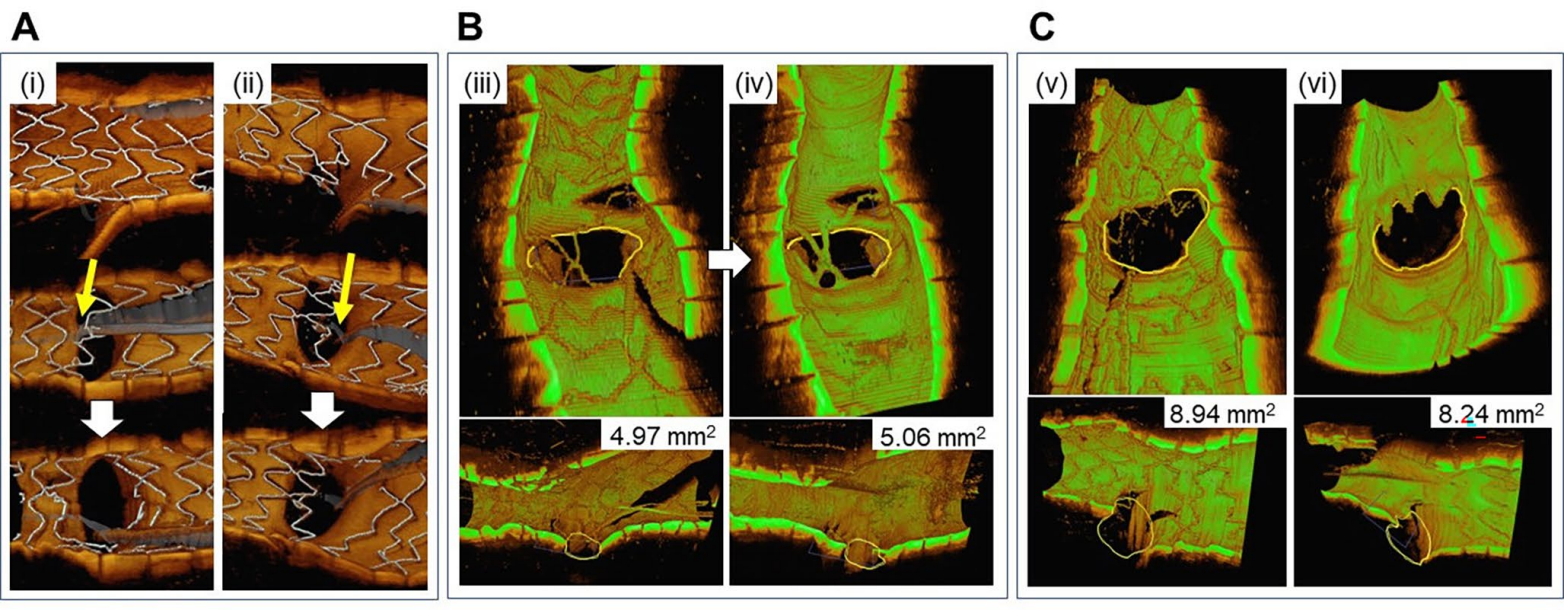
**3D-OCT assessments at the side-branch ostium. A: Rewiring position classification.***Yellow arrows* indicate rewiring wires after main-vessel stenting for a bifurcation lesion. (i) Optimal rewiring. (ii) Sub-optimal rewiring. B, C: The side-branch ostial area (SBOA) and compartments at the side branch ostium at the post-procedure and the 9-month follow-up in the left main coronary artery (LMCA). (iii and iv) *Yellow circles* indicate the SBOA measured by dedicated software (QAngioOCT^®^) post-procedure (v) and (vi) at the 9-month follow-up. The number of compartments at the side-branch ostium in panel B remained unchanged at 4, whereas the 4 compartments at the side-branch ostium changed to 1 compartment in panel C. The SBOA changes and change ratios in panel B were +0.09 mm^2^ and −0.70 mm^2^ and those in panel C were +1.8% and −7.8%, respectively

The SBOA at the post-procedure and the 9-month follow-up was measured by a cut-plane analysis that was previously validated with dedicated software (QAngioOCT; Medis Specials), in which the area enclosed by the intimal border of the side-branch manually delineated in the cut-plane view with the carina as the base, parallel to the vessel wall in the cross-sectional image, and with the smallest side-branch area in the longitudinal image was measured. The SOBA measured by cut-plane analysis of a main-vessel OCT pullback had a high correlation with measurements performed in a side-branch OCT pullback and was more accurately evaluated than the measurements by QCA analysis [9, 10]. The change ratio of the SBOA was calculated by the following formula as described previously: (SBOA at the 9-month follow-up – SBOA at the post-procedure)/SBOA at the post-procedure ×100 [4]. The number of compartments (CN) divided by the incomplete apposed stent struts at the side-branch ostium on 3D-OCT was counted at the post-procedure and the 9-month follow-up as described previously [4]. The change ratio of CN was calculated by the following formula: (CN at the post procedure – CN at the 9-month follow-up)/CN at the post-procedure ×100 (Fig. 1). All measurements were performed at the core laboratory.

### Clinical outcomes

All-cause death, cardiac death, non-fatal myocardial infarction (MI), stent thrombosis at the deployed stent site, target vessel revascularization (TVR) without target lesion revascularization (TLR), TLR, in-stent restenosis in the main vessel stenting site, and restenosis at the side-branch ostium were checked at the 9-month follow-up. In-stent restenosis in the main vessel and restenosis at the side-branch ostium were defined as > 50% stenosis compared to post-PCI values in the bifurcation segment, determined visually. Major adverse cardiac events (MACE) were defined as a composite of cardiac death, MI, TVR, and TLR at the bifurcation site.

### Statistical analyses

Continuous variables are represented as the mean ± standard deviation (SD) or the median and interquartile range (IQR) and were compared using either Student’s *t*-test for normally distributed variables or the non-parametric Wilcoxon rank-sum test for non-normally distributed variables. Two related continuous variables between the post-procedure and the 9-month follow-up were compared using Wilcoxon’s matched paired test. Categorical variables were compared using the χ^2^ test or Fisher’s exact test. A p-value of 0.05 was considered significant. The statistical analyses were performed using JMP, ver. 16 for Windows (SAS Institute, Cary, NC, USA).

## Results

### Study population and lesion and procedure characteristics

Of the 168 bifurcation lesion cases collected, the rewiring position after main-vessel crossover stenting was assessable for 106 single-stent KBI lesions. The assessment of a rewiring position by 3D-OCT was feasible regardless of the status of LMCA versus non-LMCA bifurcation, i.e., LMCA: 41 of 42 cases (97.6%); non-LMCA: 65 of 76 cases (85.5%). A guidewire shadow and a non-uniform rotation distortion made it difficult to confirm the rewiring position in 9 cases (1 LMCA and 8 non-LMCA cases) and 3 cases (no LMCA cases, 3 non-LMCA cases), respectively.

**Fig. 2:**
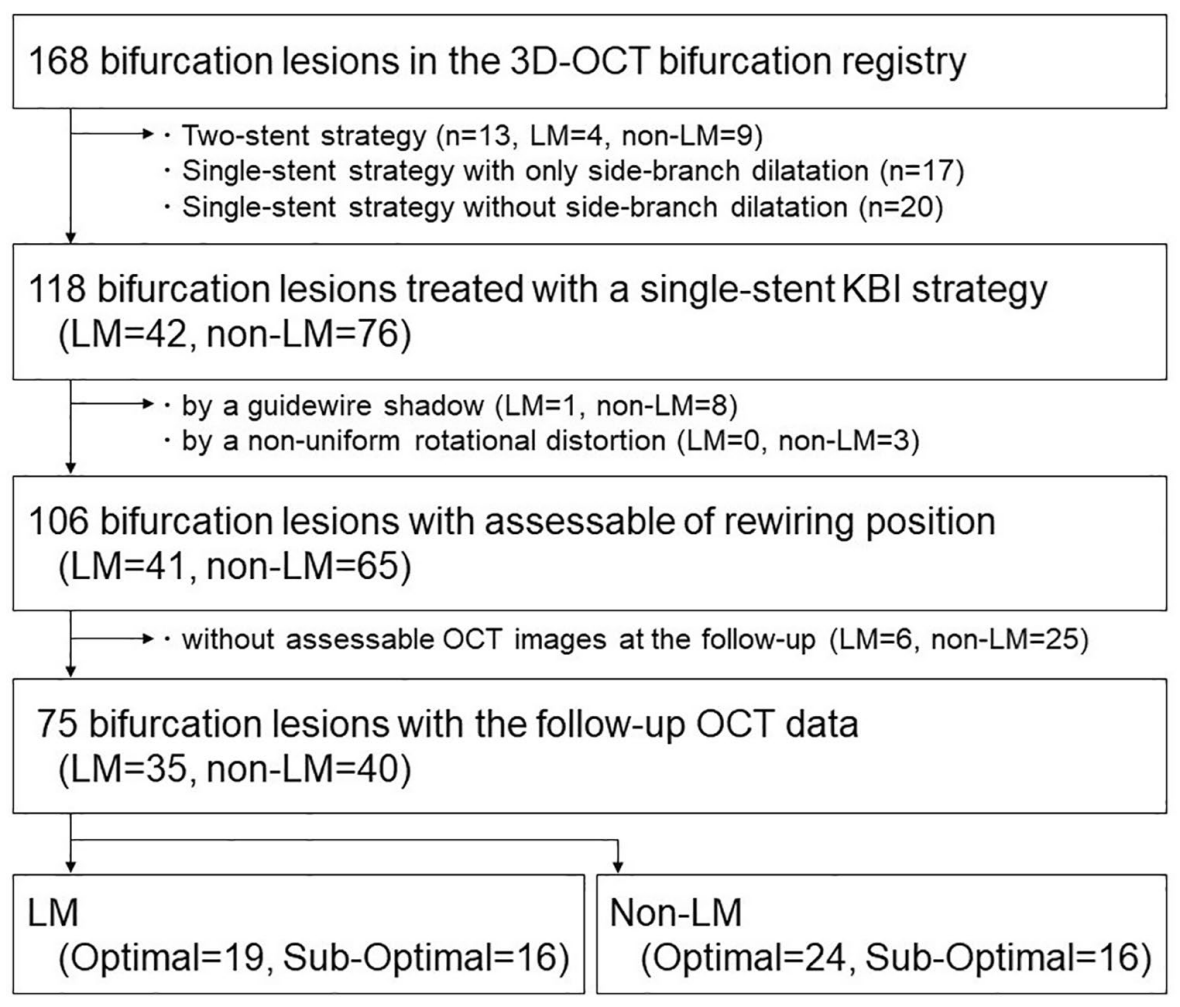
**Flow chart**. The term “optimal” means the optimal rewiring position defined as a condition that meets link-free-type and distal rewiring requirements. The term “sub-optimal” indicates non-optimal cases. Single-stent KBI is a strategy with main-vessel stenting crossing over the side branch for a bifurcation lesion. LMCA: left main coronary artery; KBI: kissing-balloon inflation; OCT: optical coherence tomography

The numbers of LMCA and non-LMCA cases with assessable OCT imaged post-procedure and at the follow-up were 35 and 40, and the numbers of cases with the optimal rewiring type in these LMCA and non-LMCA groups were 19 (54.3%) and 24 (60%), respectively (Fig. 2). The lesion and procedure characteristics of the optimal and the sub-optimal rewiring groups are shown in Table 1. The number of true bifurcation lesions in the optimal rewiring cases tended to be greater than that in the sub-optimal rewiring cases.

**Table 1:** Lesion and procedure characteristics

	LMCA			Non-LMCA		
	Optimal (n = 19)	Sub-optimal (n = 16)	P value	Optimal (n = 24)	Sub-optimal (n = 16)	P value
Age,	71.3 ± 9.0	72.4 ± 7.9	1.00	69.3 ± 10.4	74.6 ± 7.3	0.06
Male, n (%)	11 (57.9)	13 (81.3)	0.14	20 (83.3)	8 (50.0)	0.02
Hypertension, n (%)	16 (84.2)	15 (93.8)	0.38	22 (91.7)	15 (93.8)	0.81
Diabetes mellitus, n (%)	7 (36.8)	10 (62.5)	0.13	12 (50.0)	7 (43.8)	0.70
Dyslipidemia, n (%)	16 (84.2)	8 (50)	0.03	17 (70.8)	12 (75.0)	0.77
Smoker, n (%)	10 (52.6)	9 (56.3)	0.83	14 (58.3)	7 (43.8)	0.37
Serum creatinine,	0.77 ± 0.15	0.91 ± 0.27	0.15	1.01 ± 0.82	0.78 ± 0.17	0.20
Ejection fraction (%)	61.3 ± 11.1	62.3 ± 7.7	0.93	59.5 ± 11.7	60.7 ± 9.4	1.00
Primary disease, n (%)			0.07			0.35
Stable angina pectoris	11 (57.9)	10 (62.5)		11 (45.8)	11 (68.8)	
Unstable angina pectoris	4 (21.1)	0 (0.0)		1 (4.2)	0 (0.0)	
Old myocardial infarction	0 (0.0)	3 (18.8)		4 (16.7)	3 (18.8)	
Silent ischemia	4 (21.1)	3 (18.8)		8 (33.3)	2 (12.5)	
Bifurcation site, n (%)						0.08
LMCA	19 (100)	16 (100)		-	-	
LAD	-	-		18 (75.0)	8 (50.0)	
LCX	-	-		2 (8.3)	6 (37.5)	
RCA	-	-		4 (16.7)	2 (12.5)	
Medina classification, n (%)			0.07			0.17
(1, 1, 1)	4 (21.1)	2 (12.5)		7 (29.2)	1 (6.3)	
(1, 1, 0)	3 (15.8)	1 (6.3)		4 (16.7)	1 (6.3)	
(1, 0, 1)	2 (10.5)	0 (0.0)		3 (12.5)	1 (6.3)	
(1, 0, 0)	2 (10.5)	0 (0.0)		0 (0.0)	1 (6.3)	
(0, 1, 1)	2 (10.5)	0 (0.0)		4 (16.7)	3 (18.8)	
(0, 1, 0)	6 (31.6)	13 (81.3)		6 (25.0)	9 (56.3)	
True bifurcation, n (%)	8 (42.1)	2 (12.5)	0.05	14 (58.3)	5 (31.3)	0.09
2ndDES/3-links type, n (%)	2 (10.5)	1 (6.3)	0.65	6 (25.0)	6 (37.5)	0.40
Link-free type, n (%)	19 (100)	0 (0.0)	< 0.0001	24 (100)	2 (12.5)	< 0.0001
Distal rewiring, n (%)	19 (100)	13 (81.3)	0.048	24 (100)	10 (62.5)	0.001
Stent size, mm	3.1 ± 0.4	3.5 ± 0.3	0.009	2.9 ± 0.4	2.6 ± 0.3	0.03
Stent length, mm	20.3 ± 5.4	21.9 ± 7.6	0.40	24.6 ± 7.3	23.4 ± 7.1	0.49
Kissing balloon Dilatation						
MV balloon size, mm	3.3 ± 0.4	3.5 ± 0.4	0.24	3.0 ± 0.3	2.7 ± 0.3	0.03
SB balloon size, mm	2.6 ± 0.4	2.9 ± 0.4	0.009	2.2 ± 0.3	2.0 ± 0.2	0.02
Pressure, atm	9.4 ± 3.1	7.8 ± 2.6	0.10	7.5 ± 2.9	8.1 ± 2.7	0.67
POT, n (%)	12 (63.2)	6 (40.0)	0.18	10 (41.7)	5 (31.3)	0.50
3D-OCT guidance	18 (94.7)	10 (62.5)	0.018	10 (41.7)	6 (37.5)	0.79
Contrast volume, ml	145 ± 35	148 ± 38	0.77	161 ± 56	153 ± 55	0.69
Radiation time, min	44.1 ± 18.7	32.1 ± 12.0	0.05	33.4 ± 18.8	30.8 ± 16.8	0.51
Follow-up duration, days	330 ± 89	322 ± 40	0.88	300 ± 41	297 ± 42	0.99

Values are % (n) or mean ± standard deviation. MV = main vessel; LAD = left anterior descending artery; LCX = left circumflex coronary artery; LMCA = left main coronary artery; OCT = optical coherence tomography; POT = Proximal optimization technique; RCA = right coronary artery; SB = side-branch.

### QCA analysis and cross-sectional OCT image analysis

The QCA analysis detected no significant differences between the optimal and sub-optimal rewiring groups except for the reference vessel diameter of the side-branch at the pre-procedure in the LMCA group and the percent diameter stenosis of the distal main vessel at the 9-month follow-up in the non-LMCA group (Suppl. Appendix Table 1). There was no significant difference in the average of the neointima area in the proximal and distal main vessels between the optimal and sub-optimal rewiring groups in the cross-sectional OCT image analysis (Table 2). There was a significant difference in the prevalence of ISA at the side branch side between the optimal and sub-optimal rewiring cases (LMCA: 6.7% vs. 14.2%, p = 0.015; non-LMCA: 2.5% vs. 12.4%, p = 0.0004) (Table 2).

**Table 2:** Cross-sectional OCT analysis

	LM			Non-LM		
	Optimal (n = 19)	Sub-optimal (n = 16)	P value	Optimal (n = 24)	Sub-optimal (n = 16)	P value
At the post-procedure						
Average SA of PMV, mm^2^	9.86 ± 2.26	11.84 ± 2.42	0.017	7.00 ± 1.59	6.23 ± 1.51	0.16
Average SA of DMV, mm^2^	6.89 ± 1.36	7.83 ± 2.22	0.21	5.76 ± 1.57	4.86 ± 1.33	0.048
At the follow-up						
Average LA of PMV, mm^2^	9.97 ± 2.54	12.16 ± 3.98	0.12	6.48 ± 1.99	6.09 ± 1.66	0.57
Average LA of DMV, mm^2^	6.77 ± 1.32	7.14 ± 2.25	0.58	5.04 ± 1.46	4.42 ± 1.24	0.13
Average SA of PMV, mm^2^	10.31 ± 2.31	12.47 ± 3.73	0.10	6.95 ± 1.81	6.43 ± 1.66	0.52
Average SA of DMV, mm^2^	7.20 ± 1.49	7.71 ± 2.19	0.46	5.62 ± 1.41	4.80 ± 0.98	0.06
Average NIT of PMV, mm^2^	0.34 ± 0.65	0.32 ± 0.52	0.61	0.46 ± 0.80	0.34 ± 0.40	0.29
Average NIT of DMV, mm^2^	0.43 ± 0.84	0.57 ± 0.60	0.48	0.58 ± 0.64	0.37 ± 0.47	0.34
ISA in the bifurcation area*, %						
Total	10.3 (2.2, 14.3)	14.8 (6.2, 24.5)	0.12	2.9 (0.9, 6.3)	13.7 (7.2, 21.4)	0.0001
Opposite side of the side-branch	1.2 (0.0, 3.8)	0.0 (0.0, 1.7)	0.14	0.0 (0.0, 0.0)	0.0 (0.0, 3.8)	0.16
Side-branch side	6.7 (1.4, 13.1)	14.2 (6.2, 24.0)	0.015	2.5 (0.0, 5.4)	12.4 (7.2, 16.5)	0.0004

Values are % (n), % (n/N), or least square mean (95% confidence interval). DMV = distal main vessel; ISA = incomplete stent apposition; LA = lumen area; LM = left main coronary artery; NIT = neointima area; PMV = proximal main vessel; SA = stent area

### 3D-OCT image analysis

**Fig. 3:**
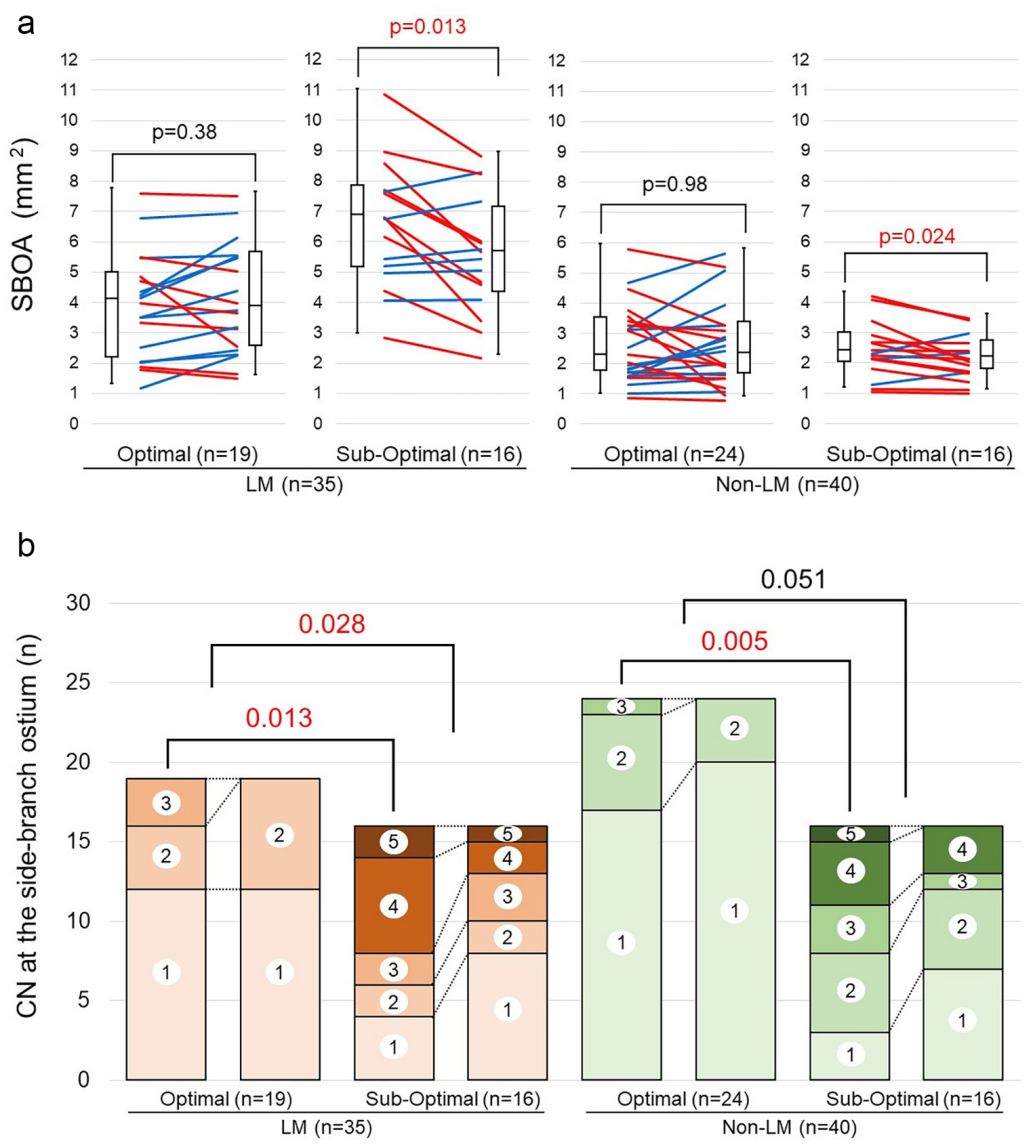
**a**: **Side-branch ostial area change in the LMCA and non-LMCA groups**. *Red lines*: the SBOA decreased. *Blue lines*: the SBOA increased. Figure 3. **b**: **Change of the number of compartments in the LMCA and non-LMCA groups.** CN: the number of compartments made by incomplete stent apposition (ISA) at the side-branch ostium

**Fig. 4:**
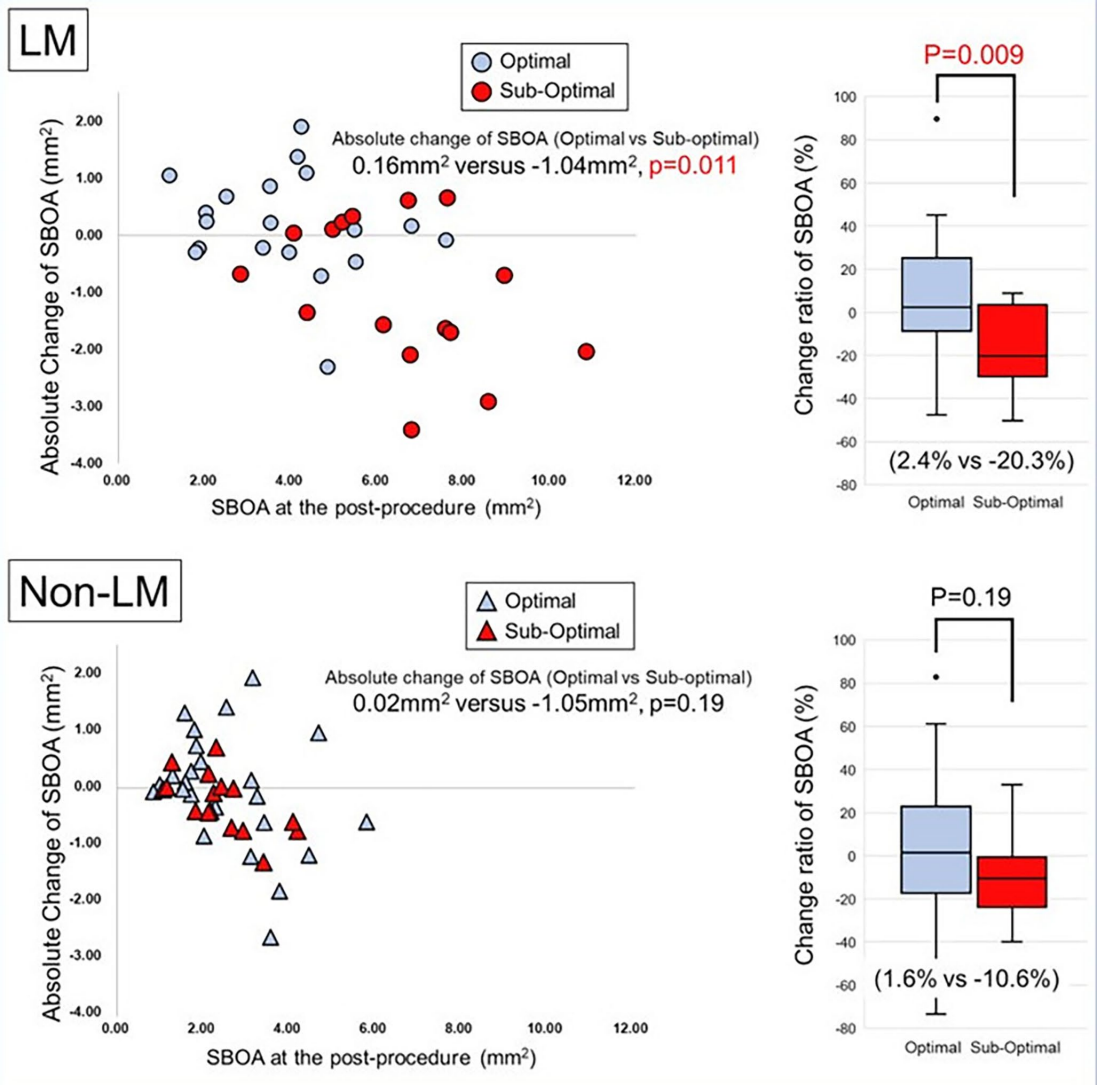
**The Relationship Between the Side-branch Ostial Area at the Post-procedure and the Absolute Change in the LMCA and non-LMCA Groups** “Optimal” indicates the optimal rewiring position, which was defined as the condition meeting the link-free-type and distal-rewiring requirements. *Blue circles*: cases of optimal rewiring in the left main coronary artery (LMCA) group. *Blue triangles*: cases of optimal rewiring in the non-LMCA group. *Red circles*: cases of sub-optimal rewiring in the LMCA group. *Red triangles*: cases of sub-optimal rewiring in the non-LMCA group. SBOA: side-branch ostial area

The SBOA with the sub-optimal rewiring position was significantly reduced compared to the SBOA with the optimal rewiring position regardless of the LMCA or non-LMCA status (Fig. 3a). There was a significant difference in the CN at the post-procedure between the optimal rewiring and sub-optimal rewiring cases in both the LMCA and non-LMCA groups. Moreover, there was a significant difference in the change ratio of the CN in the LMCA group between the optimal rewiring and sub-optimal rewiring cases (Table 3; Fig. 3b). The absolute change and the change ratio of the SBOA in the LMCA group were significantly reduced between the optimal rewiring and sub-optimal rewiring cases (0.16 mm^2^ vs. −1.05 mm^2^, p = 0.011; 2.4% vs. −20.3%, p = 0.009) (Fig. 4). There was no significant difference in the change ratio of the SBOA in the sub-optimal rewiring cases between the LMCA and non-LMCA (− 20.3% vs. −10.6%, p = 0.51).

**Table 3:** 3D-OCT analysis

	LMCA			Non-LMCA		
	Optimal (n = 19)	Sub-optimal (n = 16)	P value	Optimal (n = 24)	Sub-optimal (n = 16)	P value
Compartment number*						
Compartment number, n	1 (1, 2)	3.5 (1.25, 4)	0.013	1 (1, 2)	2.5 (2, 4)	0.005
Change ratio of the CN, %	0 (0, 0)	10 (0, 50)	0.028	0 (0, 0)	0 (0, 50)	0.051
Side-branch ostial area, mm^2^						
SBOA (Post-procedure)	3.96 (2.04, 4.84)	6.75 (5.03, 7.69)	0.0006	2.16 (16.2, 3.37)	2.28 (1.90, 2.87)	0.99
SBOA (9-month follow-up)	3.73 (2.43, 5.52)	5.54 (4.21, 7.00)	0.036	2.21 (1.54, 3.22)	2.09 (1.66, 2.59)	0.59
Absolute change	0.16 (-0.3, 0.87)	-1.05 (-1.97, 0.19)	0.011	0.02 (-0.61, 0.67)	-0.29 (-0.71, -0.01)	0.24
Change ratio of the SBOA, %	2.4 (-8.6, 25.3)	-20.3 (-29.8, 3.63)	0.009	1.6 (-17.1, 22.8)	-10.6 (-23.7, -0.7)	0.19

Values are % (n), % (n/N), or least square mean (95% confidence interval). *The CN means the number of compartments created by incomplete stent apposition at the side-branch ostium. LMCA = left main coronary artery; SBOA = side-branch ostial area

### Representative cases

**Fig. 5:**
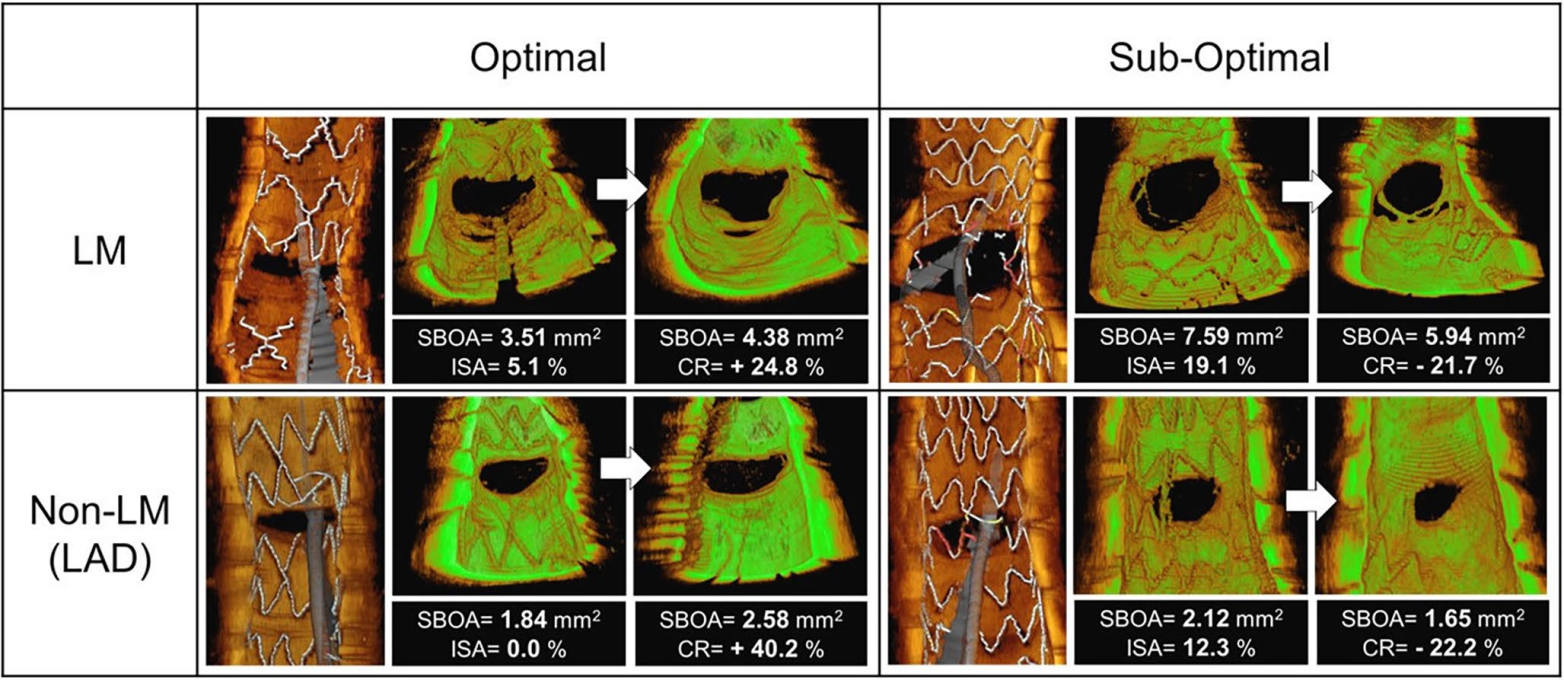
**Representative cases from each group**. The optimal wiring cases had a preserved side-branch ostial area (SBOA) at the follow-up in both the left main coronary artery (LMCA) group and the non-LMCA group, whereas the sub-optimal cases had a high rate of incomplete stent apposition (ISA) and a reduced SBOA at the follow-up. CR: the change ratio of the SBOA between the post-procedure and the follow-up

Four representative cases are shown in Fig. 5. The cases with a sub-optimal rewiring position in the LMCA group or non-LMCA group had a high prevalence of ISA at the side-branch ostium, and the SBOAs were reduced. In contrast, there was little ISA among the cases with an optimal rewiring position in the LMCA or non-LMCA group, and the SBOAs were preserved at the 9-month follow-up.

### Clinical outcomes

As shown in Table 2 in the Supplementary Appendix, there was no significant difference in MACE between the optimal and sub-optimal rewiring group regardless of in the LMCA and non-LMCA groups. ALL TLR and ISR were observed at the side branches. Regarding the restenosis of the side-branch ostium in the non-LMCA group, there was a significant difference between the optimal and sub-optimal rewiring cases: 4.2% (1/24) versus 31.3% (5/16), respectively; p = 0.019.

## Discussion

The SBOAs with the optimal rewiring position in the bifurcation lesions treated with single-stent KBI in the 3D-OCT bifurcation registry showed no significant change at the 9-month follow-up regardless of whether they were in the LMCA or non-LMCA. However, the SBOAs with the sub-optimal rewiring were reduced in both LMCA and non-LMCA cases. Especially, the change ratio of the SBOAs with the sub-optimal rewiring in LMCA was significantly reduced compared with that with the optimal rewiring. There was no significant difference in clinical outcomes between the optimal and sub-optimal rewiring cases in either the LMCA or non-LMCA group, with the exception of the in-stent restenosis assessed visually in the non-LMCA group.

### Side-branch ostial area preservation at the follow-up

Our earlier investigation demonstrated that side-branch dilatation with the optimal rewiring in patients with single-stent KBI enabled a reduction of ISA in the bifurcation area [2], and the SBOA was still expanded at 6–12 months follow-up. On the other hand, the SBOA dilated with the sub-optimal rewiring tended to be reduced at follow-up [4]. The underlying mechanism was demonstrated to be a removal of the ISA, which created small compartments at the side-branch ostium filled with tissue at 1 year after a single-stent KBI. We speculate that this mechanism is equivalent to “fenestrated” restenosis in patients without side-branch dilatation. However, the findings in our earlier investigation were based on retrospective data obtained at a single center, and the number of cases was too small to be divided into LMCA and non-LMCA groups. The optimal rewiring cases in the present study had a lower ISA rate at the side-branch side, fewer compartment numbers at the post-procedure, and more preserved SBOA at the follow-up compared to the sub-optimal cases irrespective of the LMCA versus non-LMCA status, as in our earlier investigation. These results confirmed our hypothesis that optimal rewiring identified by 3D-OCT preserved the SBOA at the follow-up.

### Optimal rewiring conditions

Optimal rewiring conditions were defined as the combination of a link-free configuration at the SB ostium and distal rewiring. The jailing configuration is currently uncontrollable, while the distal rewiring could be achieved by 3D-OCT guidance [11]. The link-free type accounted for 60% of the present cases. A previous study reported a success rate of 55–67% for distal rewiring under angio-guidance only [3]. Thus, the frequency of optimal conditions is estimated at 33–40% among cases treated with angio-guidance only. The OPTIMUM study, a randomized investigation of the impact of 3D OFDI guidance on the acute ISA after KBI compared to angio-guidance only, showed that 100% of the distal rewiring was achieved by 3D-OFDI guidance. In the present study, 57% (43/75) of all cases and 95.6% (43/45) of cases with the link-free configuration achieved the optimal condition and maintained SBOA at the 9-month follow-up. Therefore, controlling the rewiring point under 3D-OCT/OFDI guidance may be important to maintain SBOA in the chronic phase.

### The 3D-OCT guidance in LMCA bifurcation

It was reported that PCI for LMCA bifurcation lesions, which have difference anatomic and prognostic characteristics compared with non-LMCA bifurcation lesions [6], is associated with a higher risk of target lesion failure compared to PCI for non-LMCA bifurcation lesions [5]. Moreover, the use of intravascular ultrasound (IVUS) in PCIs for unprotected LMCA bifurcation lesions was reported to result in improved 12-month mortality [12], and has been recommended as CLASS 2a in the 2021 ACC/AHA/SCAI Guideline and the 2018 ESC/EACTS Guideline [13, 14]. However, OCT has not been indicated for PCIs for LMCA bifurcation lesions due to incomplete blood washout at ostial LMCA. The higher-resolution of OCT compared to IVUS can contribute to the selection of the appropriate stent and balloon size by achieving more accurate measurements of the coronary lumen size compared to IVUS and angiography [15]; the higher-resolution also permits assessment of the calcification size in order to determine the need for scoring and debulking devices, and confirmation of precise post-procedure findings that will be used to decide the procedure endpoint [16]. In addition, the use of OCT enables online 3D reconstruction and identification of the stent configuration and wire position during the procedure. OCT is expected to improve the clinical outcome of PCIs for LMCA bifurcation.

In the present study, the use of OCT in the LMCA bifurcation was feasible, although there were concerns that contrast washout would be poor due to the large-vessel diameters. In the LM group, SBOA was maintained under the optimal condition, but SBOA was decreased in the sub-optimal condition at follow-up. The 3D-OCT guidance, which can be used to select the optimal rewiring position, might avoid stent deformation by side-branch dilatation with sub-optimal rewiring.

### Clinical outcomes

We detected no significant difference in clinical outcomes between the optimal rewiring group and the sub-optimal rewiring group except for restenosis at the side-branch ostium in the non-LMCA group. In the non-LM group, small absolute change in SBOA was considered to be related to the TLR. In contrast, in the LM group, although the change ratio of SBOA was significantly reduced in the sub-optimal group, there was no significant difference in clinical events between the optimal and sub-optimal group. Several plausible explanations for this finding were considered: (1) the SBOA at FU in the sub-optimal cases remained larger than that in the optimal cases; (2) the study population was too small to evaluate clinical events; (3) event cases were not included due to lack of OCT at follow-up. There were 2 cases with a MACE among the patients without OCT data at the follow-up (n = 31) among the cases of single-stent KBI (n = 106) in the 3D-OC Bifurcation Registry. Both of these cases had sub-optimal rewiring in an LMCA bifurcation; 1 patient died and the other underwent TLR for the side branch. The results of the large multicenter and randomized Optical Coherence Tomography Optimized Bifurcation Event Reduction (OCTOBER) Trial with a sufficient sample size are eagerly awaited [17].

### Study limitations

Our present investigation was not part of a randomized study, and the decisions of whether to use and how to respond to the OCT images were left to the discretion of the operator, with no specific guidelines for optimal stenting. The types of drug-eluting stent (DES) included are another major limitation. Moreover, the instances of TLR were site-reported and not adjudicated by an independent clinical events committee. The number of cases was relatively small and thus the statistical power for assessment of clinical outcomes was insufficient; more detailed and long-term analyses are needed. Finally, additional investigations about the clinical impact of 3D-OCT in PCIs of bifurcation lesions are also warranted.

## Conclusions

In patients with single crossover stenting and kissing-balloon inflation in the 3D-OCT bifurcation registry, the SBOA dilated with the optimal rewiring position defined as distal rewiring with the link-free type was preserved at the 9-month follow-up regardless of the LMCA versus non-LMCA bifurcation status, whereas the SBOA with sub-optimal rewiring was reduced. In the LMCA cases with sub-optimal rewiring, there was a significant decrease in the SBOA at the follow-up due to tissue attachment of the small compartmented cells. The use of 3D-OCT, which can identify the stent configuration and the rewiring position, is potentially a beneficial tool in percutaneous coronary interventions of bifurcation lesions.

## Perspectives

### What Is Known?

Three-dimensional OCT can assess the stent configuration and wire position in the bifurcation area. Side-branch dilatation with link-free and distal rewiring (the optimal condition) can (*i*) reduce ISA at a bifurcation segment with single crossover stenting with kissing-balloon inflation and (*ii*) optimize the side branch.

### What Is New?

The optimal rewiring position preserved the side-branch ostial area at the follow-up regardless of LMCA or non-LMCA status, whereas the sub-optimal wiring reduced the side-branch ostial area and had more compartments at the side-branch ostium.

### What Is Next?

Further investigation using other patient cohorts with more lesions and over longer terms are warranted to validate the clinical utility of using 3D-OCT to determine the guidewire rewiring position to the side branch.

## Electronic supplementary material

Below is the link to the electronic supplementary material.

Supplementary Material 1

## Abbreviations

DES: drug-eluting stent
ISA: incomplete stent apposition
ISR: in-stent restenosis
KBI: kissing-balloon inflation
LMCA: left main coronary artery
MACE: major adverse cardiac events
OCT: optical coherence tomography
PCI: percutaneous coronary intervention
SBOA: side-branch ostial area
TLR: target lesion revascularization
