# Supplementary material for: Serial changes of the side-branch ostial area after single crossover stenting with kissing-balloon inflation
Source: Int J Cardiovasc Imaging. 2023 May 16;39(8):1593–603. doi: 10.1007/s10554-023-02853-7 (PMC10504099; doi:10.1007/s10554-023-02853-7)
Supplement: Supplementary file 1 — Supplementary Material 1 [file 10554_2023_2853_MOESM1_ESM.docx]

Table 1. QCA analysis

|  | LM | | | Non-LM | | |
| --- | --- | --- | --- | --- | --- | --- |
|  | Optimal (n=19) | Sub-optimal (n=16) | P value | Optimal (n=24) | Sub-optimal (n=16) | P value |
| Reference vessel diameter, mm |  |  |  |  |  |  |
| Proximal main vessel | 3.4 (2.9, 3.8) | 3.9 (3.4, 4.5) | 0.03 | 2.9 (2.7, 3.2) | 2.7 (2.5, 3.0) | 0.18 |
| Distal main vessel | 2.5 (2.4, 2.8) | 2.6 (2.1, 3.3) | 0.35 | 2.3 (2.1, 2.6) | 2.2 (2.1, 2.5) | 0.17 |
| Side-branch | 2.6 (2.3, 2.7) | 2.9 (2.6, 3.4) | 0.02 | 2.0 (1.7, 2.3) | 1.9 (1.6, 2.0) | 0.20 |
| Bifurcation angles, degree |  |  |  |  |  |  |
| Distal angle | 70 (54-83) | 64 (54-76) | 0.65 | 54 (38-62) | 53 (49-66) | 0.51 |
| Proximal angle | 145 (115-161) | 138 (125-153) | 0.48 | 155 (145-163) | 146 (134-162) | 0.10 |
| % Diameter stenosis, % |  |  |  |  |  |  |
| Proximal main vessel |  |  |  |  |  |  |
| pre-procedure | 29.8 (6.6-49.8) | 4.9 (1.1-22.1) | 0.11 | 27.7 (8.0-46.0) | 14.1 (3.5-50.0) | 0.46 |
| post-procedure | 4.8 (0.3-14.2) | 2.5 (0.4-3.9) | 0.25 | 6.0 (1.6-15.0) | 5.6 (1.3-10.8) | 0.73 |
| 9M follow up | 4.3 (0.1-11.2) | 2.3 (0.2-5.7) | 0.46 | 5.7 (2.9-12.0) | 6.0 (1.6-11.3) | 0.57 |
| Distal main vessel |  |  |  |  |  |  |
| pre-procedure | 37.8 (16.5-56.4) | 51.7 (35.6-63.2) | 0.16 | 47.3 (37.3-59.6) | 55.9 (44.2-69.5) | 0.26 |
| post-procedure | 9.6 (5.5-14.7) | 12.3 (7.4-25.3) | 0.13 | 14.3 (10.3-26.4) | 15.7 (8.8-24.2) | 0.99 |
| 9M follow up | 10.5 (4.6-18.1) | 12.2 (7.5-22.7) | 0.30 | 16.3 (10.1-25.0) | 12.5 (8.2-13.5) | 0.045 |
| Side-branch |  |  |  |  |  |  |
| pre-procedure | 18.5 (7.8-31.1) | 11.5 (7.9-19.5) | 0.46 | 39.2 (12.4-54.8) | 21.7 (18.7-29.2) | 0.26 |
| post-procedure | 22.4 (6.6-33.2) | 14.2 (7.4-23.1) | 0.38 | 26.5 (12.0-42.5) | 29.5 (18.3-53.4) | 0.17 |
| 9M follow up | 15.1 (6.8-27.6) | 12.8 (7.5-18.9) | 0.31 | 28.4 (19.4-42.4) | 20.2 (14.1-49.4) | 0.71 |
| Late lumen loss, mm |  |  |  |  |  |  |
| Proximal main vessel | -0.03  (-0.15, 0.07) | -0.01  (-0.04, 0.07) | 0.46 | 0.02  (-0.04, 0.08) | 0.01  (-0.21, 0.12) | 0.85 |
| Distal main vessel | 0.00  (-0.15, 0.14) | -0.03  (-0.13, 0.10) | 0.68 | 0.05  (-0.22, 0.42) | -0.10  (-0.31, 0.00) | 0.10 |
| Side-branch | 0.01  (-0.33, 0.22) | -0.01  (-0.37, 0.21) | 0.99 | 0.13  (-0.31, 0.29) | -0.05  (-0.31, 0.06) | 0.21 |
| TIMI flow grade, (<3) |  |  |  |  |  |  |
| Main vessel at the post-procedure | 0 | 0 |  | 1 | 0 |  |
| Side-branch at the post-procedure | 0 | 0 |  | 0 | 0 |  |

Distal angle means an angle between distal main vessel and side-branch. Proximal angle means an angle between proximal main vessel and side-branch. LM=left main coronary artery, TIMI=Thrombolysis in myocardial infarction.

Table 2. Clinical outcomes

|  | LMCA (n=35) | | Non-LMCA (n=40) | |
| --- | --- | --- | --- | --- |
|  | Optimal (n=19) | Sub-optimal (n=16) | Optimal (n=24) | Sub-optimal (n=16) |
| MACE +ISR +side-branch restenosis | 2 (10.5) | 1 (6.3) | 2 (8.3) | 5 (31.3) |
| All cause death | 0 | 0 | 0 | 0 |
| Cardiac death | 0 | 0 | 0 | 0 |
| Non-fatal MI | 0 | 0 | 0 | 0 |
| Stent thrombosis | 0 | 0 | 0 | 0 |
| TVR without TLR | 0 | 0 | 1 (4.2) | 0 |
| TLR | 1 (5.3) | 1 (6.3) | 1 (4.2) | 0 |
| ISR in the MV | 0 | 0 | 0 | 0 |
| TLR for side-branch | 1 (5.3) | 1 (6.3) | 1 (4.2) | 0 |
| Restenosis at the side-branch ostium | 2 (10.5) | 1 (8.6) | 1 (4.2) | 5 (31.3) |

ISR=in-stent restenosis; LMCA=left main coronary artery; MACE=major adverse cardiac events; MI=myocardial infarction; MV=main vessel; TLR=target lesion revascularization; TVR=target vessel revascularization
